# Dissecting conformational contributions to glycosidase catalysis and inhibition

**DOI:** 10.1016/j.sbi.2014.06.003

**Published:** 2014-10

**Authors:** Gaetano Speciale, Andrew J Thompson, Gideon J Davies, Spencer J Williams

**Affiliations:** 1 School of Chemistry and Bio21 Molecular Science and Biotechnology Institute, University of Melbourne, Parkville, Victoria 3010, Australia; 2 Department of Chemistry, University of York, Heslington, York YO10 5DD, United Kingdom

## Abstract

Glycoside hydrolases (GHs) are classified into >100 sequence-based families. These enzymes process a wide variety of complex carbohydrates with varying stereochemistry at the anomeric and other ring positions. The shapes that these sugars adopt upon binding to their cognate GHs, and the conformational changes that occur along the catalysis reaction coordinate is termed the conformational itinerary. Efforts to define the conformational itineraries of GHs have focussed upon the critical points of the reaction: substrate-bound (Michaelis), transition state, intermediate (if relevant) and product-bound. Recent approaches to defining conformational itineraries that marry X-ray crystallography of enzymes bound to ligands that mimic the critical points, along with advanced computational methods and kinetic isotope effects are discussed.

## Highlights

• The conformational itinerary describes the changes in sugar shape during catalysis.
• Stereoelectronic requirements for glycoside hydrolysis are discussed.
• Major and emerging approaches to define conformational itineraries are reviewed.
• New assignments of glycosidase conformational itineraries are summarized.

## Graphical abstract

**Figure:**
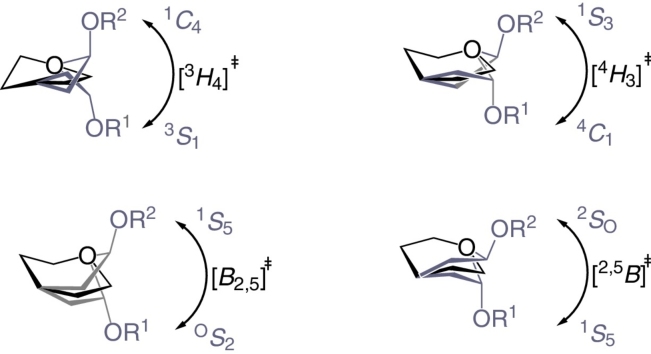

**Current Opinion in Structural Biology** 2014, **28**:1–13

This review comes from a themed issue on **Carbohydrate-protein interactions and glycosylation**

Edited by **Harry J Gilbert** and **Harry Brumer**

For a complete overview see the Issue and the Editorial

Available online 10th July 2014

**http://dx.doi.org/10.1016/j.sbi.2014.06.003**

0959-440X/© 2014 The Authors. Published by Elsevier Ltd. This is an open access article under the CC BY license (http://creativecommons.org/licenses/by/3.0/).

Glycoside hydrolases catalyze the hydrolytic cleavage of the glycosidic bond. They are enzymes of enduring interest owing to the ubiquity of carbohydrates in nature and their importance in human health and disease, the food, detergent, oil & gas and biotechnology industries. Glycoside hydrolases generally, but not quite exclusively, perform catalysis with a net retention or inversion of anomeric stereochemistry. The gross mechanisms of glycosidases were postulated by Koshland in 1953 [1^••^], and his prescient insights remain largely true to this day. The glycoside hydrolases are an immensely varied group of enzymes and are usefully classified on the basis of sequence according to the CAZy system (www.cazy.org; see also Cazypedia: www.cazypedia.org), which reveals a growing and formidable diversity of proteins (133 families as of 2014) [2]. What continues to occupy the attention of mechanistic enzymologists is a complete description of the fine details of the overall reaction coordinate. The free energy profile of catalysis is a composite of terms including: bond-making and breaking; the establishment and disbandment of stereoelectronic effects; and conformational effects. Conformational interactions include substrate-based: vicinal (e.g. eclipsing, gauche, Δ2), 1,3-diaxial, and 1,4-bridgehead; and enzyme-based: local and global conformational changes of the enzyme that occur on the time-scale of catalysis [3].

Two major areas of inquiry are active in the area of conformation and glycoside hydrolases:

1. What are the conformational changes that occur during catalysis upon substrate binding, at the transition state(s), intermediates (if relevant), and product? Aside from the elemental interest in this question, there is the potential for utilizing this information to develop glycosidase inhibitors that take advantage of the considerable amounts of energy used to selectively bind the transition state (for a glycosidase with a catalytic rate enhancement of 10^17^, the calculated transition state affinity is 10^−22^ M [4]), with the enticing possibility that differences in transition state conformation may allow the development of glycosidase-selective inhibitors.
2. Once transition-state structural information is acquired and used to inspire inhibitor development, do the resulting inhibitors actually bind by utilizing the same interactions that are used to stabilize the transition state — that is, are they genuine transition state mimics? The answers to this question speak to our abilities to realize this unique form of rational inhibitor design.

In this review we cover recent developments in the understanding of conformational reaction coordinates and how such information is acquired; and what constitutes good transition state mimicry by inhibitors. This work extends two recent comprehensive reviews [5, 6•].

## Contortions along the reaction coordinate

**Figure 1:**
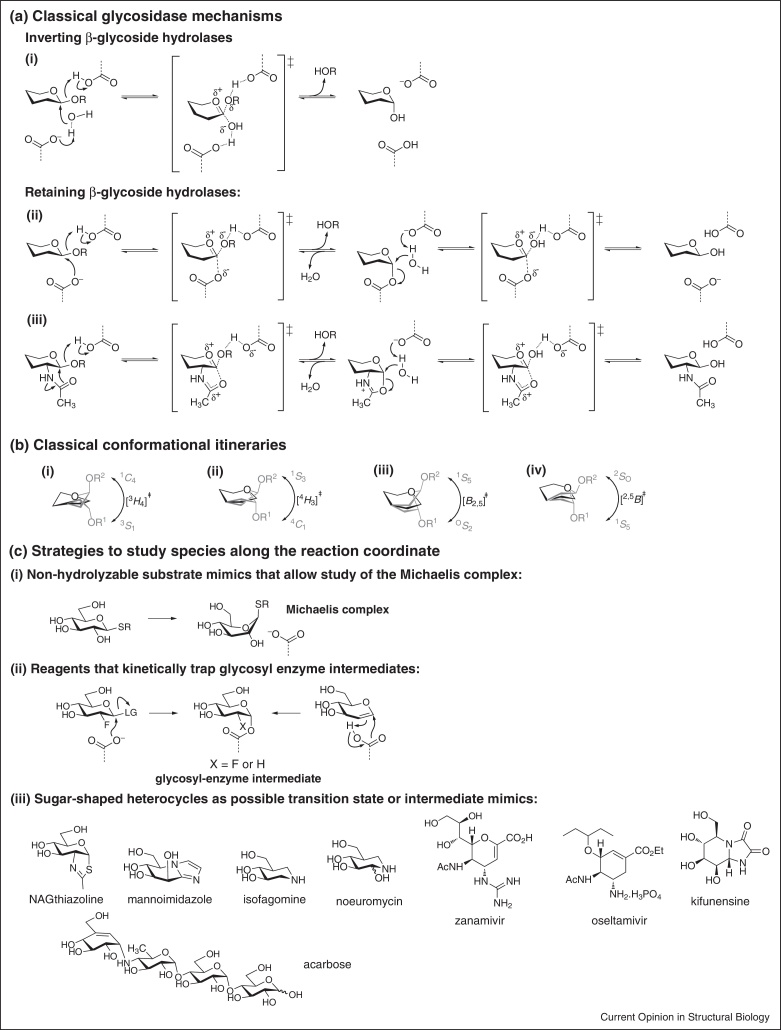
**(a)** Mechanisms of classical (i) inverting and retaining glycosidases that utilize (ii) an enzymic nucleophile or (iii) substrate-assisted catalysis. **(b)** Classical conformational itineraries around planar, oxocarbenium ion-like transition states in (i,ii) half-chair (*H*) or (iii,iv) boat (*B*) conformations. **(c)** Strategies and reagents used to study key species along the reaction coordinate.

Substantial evidence has accrued that retaining and inverting glycoside hydrolases perform catalysis through an oxocarbenium ion-like transition state with significant bond breakage to the departing group and limited bond formation to the attacking nucleophile (Figure 1a) [7]. On the basis of the four idealized half-chair and boat conformations expected for the transition state (see Side Panel A), four ‘classical’ conformational itineraries may be identified (Figure 1b). In these simplified presentations, it is apparent that C1 scribes an arc along the conformational reaction coordinate as it undergoes an electrophilic migration from the leaving group to a nucleophile. However, other ring atoms also change positions, in particular O5 and C2. The subtle change in the position of O5 has little mechanistic consequence other than to allow development of the partial double bond. Interactions at C2 are usually (but not always, see: [8]) significant and for the β-glucosidase Abg from *Agrobacterium* sp. or for α-glucosidase of *Saccharomyces cerevisiae* [9] have been shown to contribute 18–22 kJ mol^−1^ to transition state stabilization [10], highlighting that the repositioning of C2 and its substituent and other electronic changes accompanying formation of the oxocarbenium ion-like transition state can provide substantial amounts of stabilization energy. The ground state conformations and those of intermediates and transition states need not sit squarely on the graticules of the major meridians and latitudes but may be located within the conformational space nearby (see Side Panel A).

Side panel A **Theoretical considerations of the transition state conformation**

It is a stereoelectronic requirement that the development of a partial double bond between O5 and C1 results in flattening of the system C2—C1—O5—C5, with the remaining pyranose C3 and C4 atoms having freedom to move [11]. The possible idealized transition state structures that satisfy these requirements are ^3^*H*_4_, ^4^*H*_3_, ^2,5^*B* and *B*_2,5_ (and the closely related but usually higher energy ^4^*E*, ^3^*E*, *E*_4_ and *E*_3_). As the oxocarbenium ion-like transition states of glycosidases are ‘central’ transition states it is appropriate to invoke the principle of least nuclear motion, which states that elementary reactions that involve the least change in atomic position and electronic configuration will be favoured [12, 13]. Accordingly, the conformational reaction coordinate will most likely involve ground state conformations (corresponding to Michaelis, intermediate and product complexes) that are close neighbors to the transition state conformations. The conformational relationships of pyranose rings [14] may be conveniently summarized using a Cremer-Pople sphere [15] or its equivalent Mercator and polar projections (Figure I).

**Figure I:**
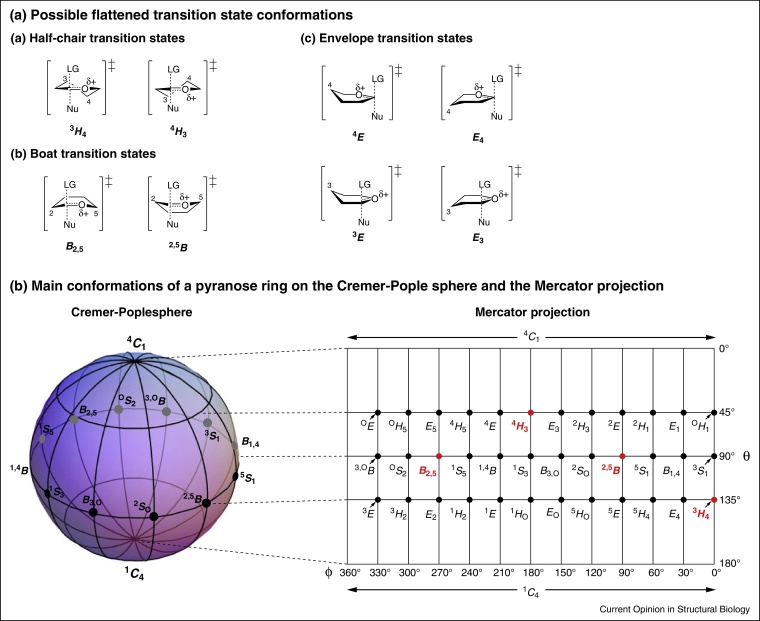

Powerful computing resources allow the calculation of the energy of every possible conformation of individual sugars providing a so-called free energy landscape (FEL). Each carbohydrate stereoisomer possesses a unique FEL, owing to the presence of various substituents, the resulting hydrogen-bonding interactions, local steric interactions, and the contribution of the anomeric effect. This computational approach was first applied to β-glucopyranose and 9 energetic minima were identified [16]. Aside from the global minimum of ^4^*C*_1_, the remaining 8 local minima were approximate *B* and *S* conformations; however these differed from the canonical *B* and *S* conformations owing to the lack of rigidity of the ring, and the presence of attractive (hydrogen bonding) and repulsive (eclipsing and 1,3-diaxial) interactions. Several of these conformations were identified as pre-activated for catalysis, with pseudo-axial C1—O1 bonds leading to a lengthening of the C1—O1 bond and a shortening of the O5—C1 bond owing to the developing anomeric effect, and partial charge at C1 (see Side Panel B). Enticingly, the pre-activated conformations are those that are most frequently observed in so-called Michaelis complexes (representing E.S complexes) studied by X-ray crystallography for GHs that process β-glucosides. FEL analysis has been extended to include α-glucopyranose, β-xylopyranose, β-mannopyranose, and β-*N*-acetylglucopyranosamine [17], and α-mannopyranose [18^••^], with the data supporting the conclusion that for these sugars the catalytically relevant conformations are frequently the energetically predisposed distorted structures. These observations are harmonious with the suggestion of Wolfenden that the fact that many enzymes achieve rates approaching the diffusion limit suggests that they have evolved to recognize species that are reasonably populous in solution [19].

Side panel B **On substrate distortion and Michaelis complexes**

The concept of substrate distortion upon binding to an enzyme in the enzyme-substrate (Michaelis) complex has long been proposed since the earliest X-ray crystallographic data of glycosidases became available. Four major principles are invoked to justify the need for a substrate to distort from a ground-state and less reactive conformation to a distorted and typically higher energy conformation: (1) the principle of least nuclear motion, which favours distorted conformations that require less nuclear movement to achieve the transition state (see Side Panel A); (2) the geometric demand that nucleophilic attack on the anomeric centre needs to be ‘in line’ with the departing nucleophile, which arises from the stereoelectronic requirement that the electrons derived from the nucleophile will populate the σ* orbital of the glycosidic bond; (3) the stereoelectronic requirement for development of a partial double bond at the oxocarbenium ion-like transition state, which requires electron donation by a suitably located n-type lone pair on the endocyclic oxygen; and (4) the fact that general acid catalytic residues of glycosidases are located within the plane of the ring, either syn or anti to the C1—O5 bond [20]. Although often invoked as a rationale for substrate distortion, Sinnott has persuasively argued that the ‘antiperiplanar lone pair hypothesis’, viz. that sp^3^ lone pairs of electrons on a heteroatom direct the departure of a leaving group from an adjacent tetrahedral carbon centre, requires implausible contortions of the pyranose ring [21].

## Defining the conformational coordinate: Is seeing believing?

X-ray crystallography is a powerful technique that provides a detailed molecular description of the catalytic machinery of an enzyme. While unliganded (apo) structures provide some information that can be used to help understand mechanism, the acquisition of complexes, with substrates (or substrate analogues), sugar-shaped inhibitors, mechanism-based inhibitors, or products have the potential to reveal intricate details of the amino acid residues involved in catalysis and the conformations of enzyme-bound species (Figure 1c). Three main strategies for the acquisition of Michaelis (E.S)-like complexes of retaining and inverting glycosidases are: firstly, co-crystallization of non-hydrolyzable substrate mimics with wildtype enzyme (Figure 1c(i)) [22], or secondly, co-crystallization of substrate with catalytically inactive mutant enzymes, or thirdly, co-crystallization of substrate and wildtype enzyme at a pH at which it is inactive. Occasionally, Michaelis complexes have been obtained serendipitously at pH values under which the enzyme is active; the reason for lack of hydrolysis in these cases is unclear [23, 24].

For retaining glycoside hydrolases that proceed through a glycosyl enzyme intermediate, fairly effective methods have been developed to allow the trapping of kinetically competent intermediates [25]; the general principle is to rapidly access the glycosyl enzyme intermediate using a good leaving group, but to modify the sugar such that its turnover to product is slowed, allowing its accumulation and study. The initial work by Legler involved addition to glycals to generate 2-deoxyglycosyl enzymes [26] or use of aryl 2-deoxyglycosides [27], and was elegantly extended by Withers to 2-deoxy-2-fluoro-, 2-deoxy-2,2-difluoro- and 5-fluoro glycosyl fluorides (and closely related 2,4-dinitrophenyl, and other activated glycosides) [25] (Figure 1c(ii)). For reasons that are not entirely clear, for α-glycosidases the use of C5-inverted 5-fluoro-glycosyl fluorides for the corresponding enzymes usually yield better trapping results than for the stereochemically-matched alternative. For retaining enzymes that proceed by anchimeric assistance from a 2-acylamido group, sulfur mimics of the proposed oxazoline (or oxazolinium ion) intermediate, most notably NAGthiazoline [28, 29], have proved effective as inhibitors and informative as mechanistic probes for crystallographically studying the intermediate (Figure 1c(iii)).

It is important to recognize that all X-ray structures of protein–ligand complexes are by their very nature not catalytically competent and thus care must be taken in how to interpret the important clues they provide in the proposal of a credible conformational itinerary. Kinetically trapped species recapitulate the major bond-forming and breaking events but the structural modifications made to allow kinetic trapping may perturb substrate interactions that are important for defining the conformational reaction coordinate. Occasionally, crystallization efforts at non-optimal pH have led to the acquisition of apparently bonafide Michaelis complexes; however even these constitute complexes with catalytically-incompetent enzymes and the interpretation of these structures must recognize that these do not lie on the reaction coordinate. Complexes with substrate and enzyme may be ‘pre-Michaelis’ complexes that represent enzyme-bound species that precede the formation of the true Michaelis complex, or may be catalytically irrelevant species that are actively misleading. Product bound to enzyme may have relaxed from its first formed conformation as the lack of a sizeable anomeric substituent prevents the enzyme from utilizing +1 subsite interactions to stabilize its conformation. Nonetheless, with some exceptions, most pseudo-Michaelis, glycosyl enzyme intermediate or thiazoline intermediate, and product complexes are sufficiently akin to hypothetical bonafide species on the reaction coordinate to allow cautious but probably reasonable insights into mechanism.

**Figure 2:**
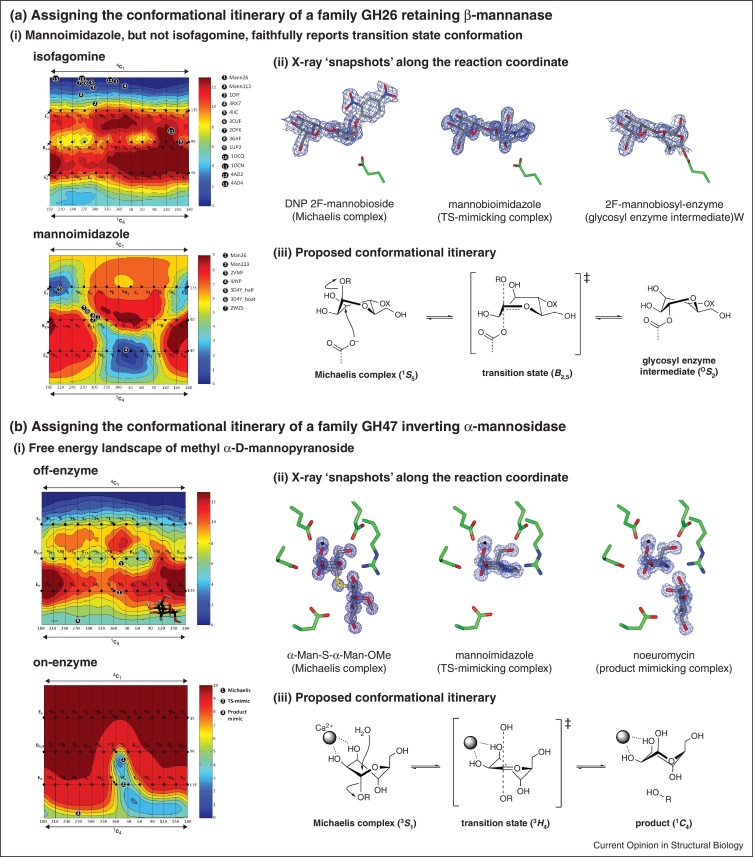
Computational studies, in concert with X-ray crystallography and inhibitor design and synthesis, assist in assigning conformational itineraries. **(a)** Assigning the conformational itinerary of *Cellvibrio japonicas* GH26 β-mannanase Man26C. (i) Free energy landscapes reveal mannoimidazole, unlike isofagomine, is able to attain the conformations relevant to glycosidase catalysis; (ii) X-ray structures of a Michaelis complex (1GVY), glycosyl enzyme intermediate (1GW1), and transition state mimicking β-mannosyl-1,4-mannoimidazole complex (4CD5); (iii) proposed conformational itinerary. **(b)** Assigning the conformational itinerary of *Caulobacter* strain K31 GH47 α-mannosidase. (i) Free energy landscapes highlight substrate preactivation off-enzyme, and reshaping of the available conformations on-enzyme; (ii) X-ray structures of Michaelis complex (4AYP), transition state mimicking mannoimidazole complex (4AYQ), and product mimicking noeuromycin complex (4AYR); (iii) proposed conformational itinerary.

Caution must also be exercised when studying complexes with sugar-shaped heterocycles that function as competitive inhibitors (Figure 1c(iii)). While superficially these compounds resemble aspects of the proposed transition state, there are intrinsic limitations of what can be mimicked in a chemically stable compound, including hybridization changes, partial charge development, and fractional bond orders. A study of the FELs of two inhibitors that display superficial transition state mimicry: isofagomine and mannoimidazole revealed dramatic differences (Figure 2a) [30^••^]. Isofagomine is strongly biased toward a ^4^*C*_1_ conformation, with potential transition state mimicking ^4^*H*_3_ and *B*_2,5_ conformations lying 12 and 8 kcal mol^−1^ higher, respectively, and importantly with a significant barrier to attaining those conformations. On the other hand while mannoimidazole prefers ^4^*H*_3_ and ^3^*H*_4_ conformations (with a 1 kcal mol^−1^ preference for the latter), the *B*_2,5_ conformation is also energetically accessible. Overlaying the observed conformations of isofagomine-type and mannoimidazole-type inhibitors from X-ray structures with mannose-processing enzymes of various GH families reveals all isofagomine complexes adopt a ^4^*C*_1_ conformation, whereas for mannoimidazole the *B*_2,5_ conformation is observed on enzymes of families GH2, 26, 38, 92 and 113, implying a ^1^*S*_5_→*B*_2,5_^‡^→^O^*S*_2_ conformational itinerary. One interesting footnote is that non-ground state conformations of isofagomine-type inhibitors have been observed on family GH6 cellulases in either ^2,5^*B*/^2^*S*_O_ or ^2^*S*_O_ conformations [31, 32]. The energetic difficulties in attaining such conformations highlight their special significance when seen and in these cases they reflect the proposed ^2^*S*_O_→^2,5^*B*^‡^→^5^*S*_1_ itinerary.

While the FEL of an isolated carbohydrate is often biased toward those conformations pre-activated for catalysis, further substrate distortion presumably occurs upon binding to enzyme. Quantum mechanics/molecular mechanics calculations of α-mannopyranose revealed that a FEL determined within the constraints of a GH47 α-mannosidase is moulded by the enzyme to dramatically limit the conformations accessible by the substrate to a previously inaccessible region of the FEL for the substrate off-enzyme (Figure 2b) [18^••^]. In support of the theoretical predictions, X-ray analysis of ‘snapshot’ complexes of the enzyme with a substrate analogue, transition state, and product mimics supported a ^3^*S*_1_→^3^*H*_4_^‡^→^1^*C*_4_ conformational itinerary predicted on the basis of the FEL remodelling.

## Sugars getting into shape: News dispatches from the families

Table 1 summarizes well-defined conformational itineraries for a range of GH enzymes (see Supporting Information for a more detailed listing). We present a few highlights from the last two years that are not covered in detail elsewhere.

**Table 1:** Conformational itineraries around various transition state conformations. Listed are families for which strong evidence in support of a conformational itinerary is available. For a more comprehensive listing see Supporting Information Table S1

Transition state conformation	GH families	Configuration of substrate	Enzymatic activities
^3^*H*_4_^‡^ (^4^*H*_5_^‡^ for sialidases)	29, 33, 34, 47	l-*fuco* (=l-*galacto*) sialic acid,	α-Fucosidase
		d-*manno*	α-Mannosidase
^4^*H*_3_^‡^	1, 2, 3, 5, 7, 10, 12, 16, 20, 22, 26, 27, 30, 84	d-*gluco*/d-*manno*	β-Glucosidase/cellulase/lichenases/b-mannosidase
		d-*galacto*	β-Galactosidase
		d-*gluco*	α-Glucosidase/α-glucanase
		d-*xylo*	β-Xylosidase/xylanase
		d-*gluco/galacto*	β-Hexosaminidase
^2,5^*B*^‡^	6, 8, 11	d-*gluco*	β-Glucosidase, chitinase
		d-*xylo*	Xylanase
*B*_2,5_^‡^	2, 26, 38, 92, 113	d-*manno*	β-Mannosidase, β-mannanase, α-mannosidase
^4^*E*^‡^	117	3,6-anhydro-l-*galacto*	α-1,3-l-Neoagarobiase

β-Hexosaminidases of family GH3 perform catalysis through a two step mechanism with the initial substitution step occurring by an enzymatic nucleophile to afford a glycosyl enzyme intermediate (Figure 1a(ii)). Insight into the reaction coordinate has been obtained through trapping a glycosyl enzyme intermediate in a ^4^*C*_1_ conformation using the mechanism based inhibitor 5F-GlcNAcF, suggesting a ^1^*S*_3_→^4^*H*_3_^‡^→^4^*C*_1_ itinerary [33^•^]. Interestingly, a complex with an inactive mutant with substrate, and of wild-type with product, also revealed ^4^*C*_1_ conformations. In this case there is strong evidence that the complex with substrate is not a bonafide Michaelis complex as a loop containing the putative histidine general acid/base undergoes a dramatic movement. In the product complex it appears that the sugar has relaxed to a more stable conformation.

**Figure 3:**
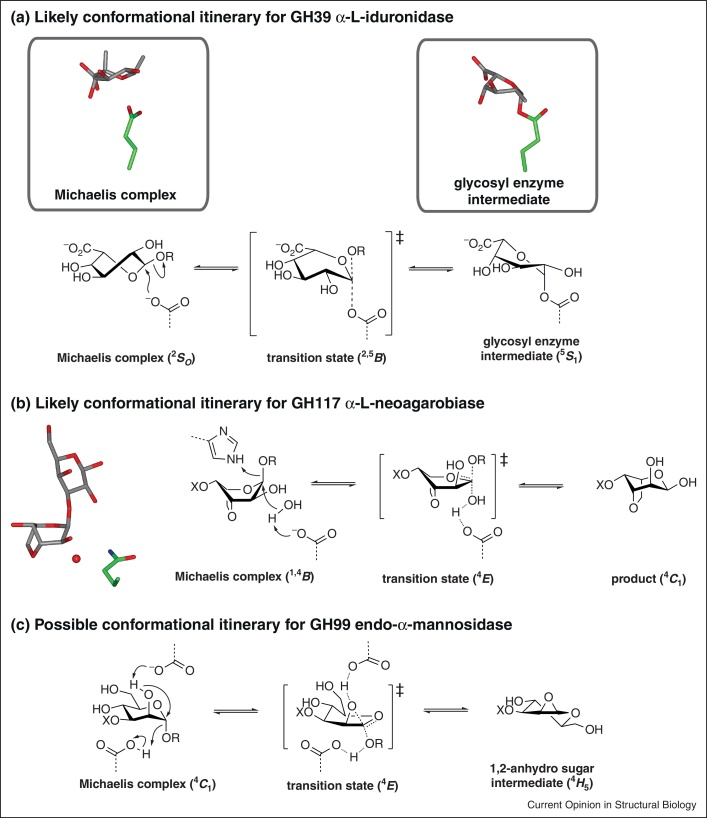
New glycosidase conformational assignments. **(a)** A likely conformational itinerary for the GH39 human α-l-iduronidase based on structures of a Michaelis complex (4KGJ) and a glycosyl enzyme intermediate (4KH2). **(b)** A likely conformational itinerary for an α-l-neoagarobiase based on a Michaelis complex (4AK7). **(c)** A possible conformational itinerary for a GH99 endo-α-mannosidase based on a proposed mechanism that proceeds through a 1,2-anhydro sugar intermediate.

Family GH39 α-l-iduronidase (IDUA) is a retaining lysosomal enzyme that assists in the stepwise degradation of heparin sulfate and dermatan sulfate, and which is of interest for enzyme replacement therapy of the associated lysosomal storage disorder (LSD) mucopolysaccharidosis type I [34^•^]. Michaelis complexes with iduronate analogues in ^2^*S*_O_ conformations, and the trapping of a glycosyl enzyme on IDUA in a ^5^*S*_1_/^2,5^*B* conformation using 2-deoxy-2-fluoro-α-l-idopyranosyluronic acid fluoride, imply a ^2^*S*_O_→^2,5^*B*^‡^→^5^*S*_1_ conformational itinerary (Figure 3a) [35^••^].

Family GH59 β-galactocerebrosidase (GALC) degrades glycosphingolipids and its deficiency leads to another LSD, Krabbe disease. X-ray ‘snapshots’ of a Michaelis complex (using 4-nitrophenyl β-d-galactopyranoside), a 2-deoxy glycosyl enzyme (from galactal addition), and product (with galactose) showed the sugar ring in a ^4^*C*_1_ conformation in all structures [36^•^]. This surprising result, reminiscent of that seen with the GH2 β-galactosidase LacZ [37], was interpreted as suggesting that no distortion of the ring occurs along the reaction coordinate. Interestingly, the acid/base residue in the substrate complex is incorrectly positioned suggesting that this is not a Michaelis complex; additionally, the product complex may have relaxed from its first-formed conformation.

Family GH117 α-1,3-l-neoagarobiase has been described as a keystone enzyme owing to its role in agarose degradation, which provides the capability for the human gut microbiota to degrade seaweed diets [38^••^]. These interesting (probably inverting) enzymes act on 3,6-anhydro-α-l-galactosides (Figure 3b). The 3,6-anhydro bridge imparts significant rigidity on the sugar to prefer ^4^*C*_1_ and ^1,4^*B* conformations. A Michaelis complex of an inactive mutant with neoagarobiose highlighted a histidine residue as a potential catalytic general acid and revealed a ^1,4^*B* conformation, suggestive of a ^1,4^*B*→^4^*E*^‡^→^4^*C*_1_ conformational itinerary.

GH11 xylanases are an as yet unresolved case. Early structures of intermediate complexes trapped with 2-fluoro sugars were interpreted as ^2,5^*B* conformations [39, 40], suggesting a possible ^2^*S*_O_→^2,5^*B*^‡^→^5^*S*_1_ itinerary. Very recently a long sought apparent Michaelis complex of xylohexaose bound to the xylanase XynII from *Trichoderma reesi* revealed a slightly distorted ^4^*C*_1_ conformation [41]. Confounding this issue, product complexes with GH11 enzymes reveal a range of different conformations that are inconsistent with the proposed itinerary.

## On the importance of being mannose

The majority of common, naturally occurring sugars in their ground-state chair conformation either have an equatorial hydroxyl at C2 (galactosides, glucosides, xylosides, fucosides), or no substituent (sialosides; formally C3). Mannosides and rhamnosides, bearing axial 2-hydroxyls in the ground state conformations, provide exceptions that have interesting and significant consequences for reactivity that lies at the heart of what has been described the recalcitrant chemistry of mannose. For α-mannosides in a ^4^*C*_1_ conformation, in addition to the existence of the stabilizing anomeric effect that dissuades substrate distortion, the presence of the strongly electron-withdrawing OH at C2 results in opposing dipoles at C1 and C2 that provide additional ground state stabilization. For β-mannosides in a ^4^*C*_1_ conformation, the anomeric effect provides little stabilization. In addition, other destabilizing effects are operative. Collectively, these can be described as a Δ2 effect, a term first coined by Reeves [42]. The Δ2 effect describes the destabilizing effect of an oxygen on one carbon that bisects two oxygens substituted on an adjacent carbon, aligning dipoles and causing gauche–gauche interactions between the vicinal oxygens. Nucleophilic substitutions at C1 of α-mannosides have to contend with a developing Δ2 effect. Reflecting these complexities, nature has devised some remarkable strategies for enzymes to solve these problems.

α-Mannosidases of families GH38, 47 and 92 are metal dependent, with crystallographic evidence for the divalent metal cation (Zn^2+^ or Ca^2+^) binding O2 and O3. This interesting observation may provide a means to overcome the high stability of the unreactive ^4^*C*_1_ conformation of α-mannosides, and encourage contraction of the ground state O2—C2—C3—O3 torsion angle within the ^4^*C*_1_ conformation of 60° toward the 0–15° angle expected at the *B*_2,5_ transition state [43]. In addition the flexible coordination number and geometry of calcium may allow coordination and delivery of the nucleophilic water, thereby providing a way to overcome a developing Δ2 effect [43]. A computational study of a GH38 α-mannosidase suggested that Zn^2+^ coordination may stabilize charge that develops on O2 at the oxocarbenium ion like transition state [44]. Remarkably, this calculation revealed that the charge on zinc varies reciprocally with the charge developing on the oxocarbenium ion-like TS.

There is now compelling evidence that family GH2, 26 and 113 retaining β-mannosidases, and GH38 retaining and GH92 inverting α-mannosidases, utilize *B*_2,5_ transition states with Michaelis complexes in a ^1^*S*_5_ (for β-) or ^O^*S*_2_ (for α-), and thus operate through ^1^*S*_5_↔*B*_2,5_^‡^↔^O^*S*_2_ conformational itineraries [30^••^]. The Michaelis complex conformation provides a pseudo axial arrangement of the anomeric leaving group and permits inline attack of the nucleophile; and importantly the ^1^*S*_5_ conformation relieves the Δ2 effect. One question that logically arises from studies of transition state conformation is whether all enzymes within a family operate with the same conformational itinerary? Family GH26 contains enzymes that act on β-mannosides, β-glucosides and β-xylosides: lichenases (which hydrolyse the mixed linkage β-1,3-; β-1,4-glucan lichenan), β-mannanase and β-1,3-xylanases. Studies of Michaelis complexes and trapped glycosyl enzymes provides good evidence for an alternative ^1^*S*_3_→^4^*H*_3_^‡^→^4^*C*_1_ itinerary for lichenases [45] and β-1,3-xylanases [46^•^]. The different conformations of the transition state of the d-*gluco*/d-*xylo* and d-*manno* configured substrates result in the substituents at C2 being pseudo-equatorial in both cases and lying at essentially the same place in space, explaining how the conserved catalytic machinery of different GH26 family members can tolerate differently configured sugars, with the specificity arising from a large difference in the positions of the C3 substituents [45], a relationship which is highlighted by the common inhibition of β-mannosidases and β-glucosidases by isofagomine lactam [47].

Uncertainty surrounds the conformational itineraries of α-mannosidases of families GH76, 99 and 125. No complexes are available for GH76 that could provide any insight into a possible itinerary. For GH99, which contains retaining endo-acting α-mannosidases, the only complexes available are with isofagomine and deoxymannojirimycin-derived inhibitors, and these bind in ^4^*C*_1_ conformations which match the ground state of the inhibitors, so it is not clear whether these complexes represent enzyme-induced or substrate-biased conformations. However, on the basis of an inability to identify a catalytic nucleophile in the complex with α-glucosyl-1,3-isofagomine, a neighboring group participation mechanism for GH99 was suggested that proceeded through a 1,2-anhydro sugar [48^•^]. This proposal implies the intermediate adopts a ^4^*H*_5_ conformation, and least nuclear motion would predict a ^4^*C*_1_→^4^*E*^‡^→^4^*H*_5_ itinerary (Figure 3c). For the inverting GH125 α-mannosidases, a pseudo Michaelis complex is available which has the −1 sugar in an undistorted ^4^*C*_1_ conformation, which matches that observed with a complex with deoxymannojirimycin [49]. The lack of distortion for enzyme bound to the non-hydrolyzable substrate is surprising.

## Neuraminidases: of conformational itineraries and transition state mimicry by inhibitors

**Figure 4:**
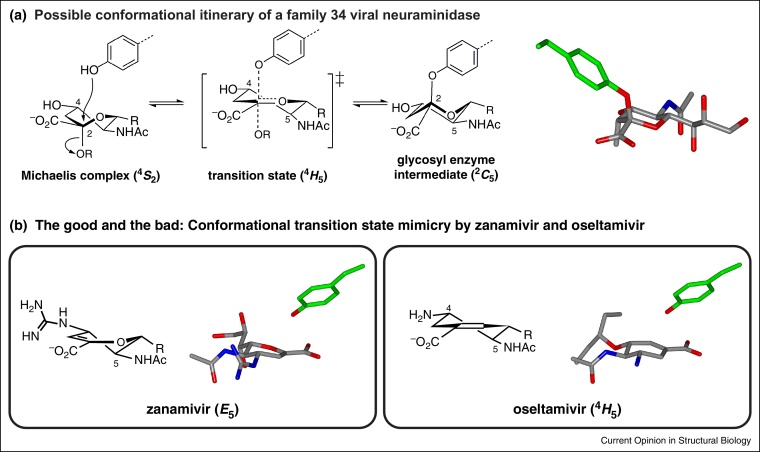
Conformational itinerary of influenza GH34 neuraminidases and conformational transition state mimicry by inhibitors. **(a)** The conformational itinerary of influenza GH34 neuraminidases informed by an X-ray structure of a glycosyl enzyme intermediate (4H52). **(b)** Complexes of anti-influenza drugs with influenza neuraminidases reveals that oseltamivir (2HU4), unlike zanamivir (1NNC), provides good conformational mimicry of the proposed sialidase transition state.

Neuraminidases (sialidases) are glycosidases that cleave sialic acid residues, with the family GH34 viral surface, retaining neuraminidases being significant as the eponymous enzymes in the HXNY classification system of influenza viruses. Influenza virus neuraminidases play key roles in the infection of cells by the virus and the ability of progeny virions to detach from an infected cell and infect new cells. In two related studies, Withers and co-workers [50^••^] and Gao and co-workers [51^•^] designed a series of neuraminidase inhibitors that combine features of the deoxyfluorosugar inhibitors modified to incorporate structural features of the clinically-approved drugs zanamivir (Relenza) and oseltamivir (Tamiflu). X-ray structures of an elusive tyrosyl enzyme intermediate revealed a ^2^*C*_5_ conformation (Figure 4a). While a ^4^*S*_2_→^4^*H*_5_^‡^→^2^*C*_5_ (equivalent to a ^3^*S*_1_→^3^*H*_4_^‡^→^1^*C*_4_ for a hexopyranose) is consistent with this data and was proposed for neuraminidases of GH33 [52], Bennet reported kinetic isotope effect analysis of the GH33 *Micromonospora viridifaciens* sialidase that implied a Michaelis complex in a ^6^*S*_2_ (^5^*S*_1_ for aldose) conformation [53], a conformation also seen in the Michaelis complex of a GH33 transialidase from *Trypanosoma cruzi*, and consistent with a ^6^*S*_2_→^4^*H*_5_^‡^→^2^*C*_5_ (equivalent to a ^5^*S*_1_→^3^*H*_4_^‡^→^1^*C*_4_ for an aldose) conformational itinerary [52].

Zanamivir and oseltamivir are potent competitive inhibitors of viral neuraminidases and bear some similarity to the proposed transition state of neuraminidase, yet it is not clear whether either compound achieves its potency through transition state mimicry. As espoused by Wolfenden [54] and Thompson [55] and elegantly summarized by Bartlett [56], critical analysis of transition-state mimicry can be achieved by comparing the effects of equivalent structural perturbations on the affinity of the true transition state (via effects on substrate *k*_cat_/*K*_M_) and on the affinity of the transition state analogue (via *K*_I_ values), plotted as a linear free energy relationship. Two approaches may be used to introduce perturbations: firstly, modifications to the inhibitor and the corresponding substrate and measurement of kinetic parameters with the wild-type enzyme, or secondly, mutation of enzymatic active site residues to afford mutant enzymes, which are studied with the same inhibitor and substrate. A limitation of the former method is the effort that needs to be expended on synthesis of derivatives, but which allow atomic level modifications to be made limited only by the imagination and synthetic chemistry. Limitations of the latter include the rather blunt tool of site-directed mutagenesis which is restricted to the genetically encoded natural amino acids, and the possibility that mutational perturbations may affect the fundamental reaction mechanism of the enzyme.

Zanamivir was designed to improve the known sialidase inhibitor neuraminic acid glycal (Neu5Ac2en) by the rational inclusion of an enzyme-specific guanidine group targeting a negatively charged pocket near the active site [57]. Neu5Ac2en bears some similarity to the transition state by virtue of *sp*^2^ hybridization at C2. It can be argued that a transition state mimicking inhibitor has the potential to provide potent inhibitors that should be resistant to mutations within the active site, as mutations that affect the ability of the inhibitor to bind should also affect the catalytic proficiency of the enzyme to similar degrees, resulting in loss of fitness for the virus. By making alterations in the structure of zanamivir at the 4-position and relating the effects of these changes upon inhibitor *K*_I_ values to the equivalent changes to the substrate and their effect upon *k*_cat_/*K*_M_ or *K*_M_ Bennet and co-workers showed that zanamivir is not a transition state analogue and is better considered a ground state analogue [58^•^]. Notably, this is confluent with the observation that influenza strains resistant to zanamivir possess reduced binding avidity for this drug but still possess catalytic competence. With impressive foresight, this possibility was suggested in the earliest publication describing the invention of zanamivir [57]. The failure of zanamivir in this Bartlett analysis is perhaps not overly surprising. Zanamivir has a double bond between C2=C3, and cannot adopt the ^4^*H*_5_ conformation predicted for the transition state of GH33 sialidases; indeed an *E*_5_ conformation is observed for zanamivir in complexes (Figure 4b). On the other hand oseltamivir (Tamiflu) is a carbocycle with a double bond located at the appropriate position to mimic the partial C2—O5 double bond at the transition state, and is observed to bind to sialidases in a ^4^*H*_5_ conformation matching that of the transition state [59]. It will be interesting to see if Bartlett analysis applied to oseltamivir provides evidence of transition state mimicry. This situation is worth comparing with the powerful α-glucosidase inhibitor acarbose, which has a double bond C5=C6 (using pyranose numbering) and cannot adopt a planar conformation matching that expected for a glycosidase transition state; Bartlett analysis of acarbose with a GH14 cyclodextrin glycosyltransferase gives good correlation of log *K*_I_ with log *k*_cat_/*K*_M_, but also good correlation with log *K*_M_ suggesting both substrate and transition state mimicry [60].

## Conclusions

A sophisticated view of glycoside hydrolase catalysis is now evident in which conformational changes occur that predispose substrates to react through oxocarbenium ion like transition states that are in accord with stereoelectronic and least nuclear motion principles. The challenges of these studies include the fact that X-ray crystal structures in complex with ligands by their very nature result in perturbation of the system for species nominally on or near the reaction coordinate, and for species off the reaction coordinate, great care needs to be taken to ensure that ground state conformational preferences do not bias interpretations. Kinetic isotope effect measurements and computational analysis can provide much needed help in assigning conformational itineraries. Compelling data is now available to assign conformational itineraries for a large number of GH families, yet as highlighted above, there are examples in which crystallographic data alone do not allow proposal of conformational itineraries. In these cases application of KIE analyses and theoretical approaches may help reveal a likely itinerary.

There is a growing need for glycosidase inhibitors that exhibit selectivity against specific glycosidases, both to enable chemical biology approaches in glycobiology such as unravelling the roles of specific glycoside hydrolases in complex biochemical pathways [61], and in translational applications, for example, as folding chaperones for treatment of lysosomal storage disorders [62], and as enzyme inhibitors targeting aberrant glycosylation [63]. One of the long-term goals of conformational analysis of the glycosidase reaction coordinate is the hope that such information can inform the design of potent inhibitors, and in addition that these may be specific for particular conformational itineraries. While using such information in the design of inhibitors is not the primary focus of this review it is probably fair to say that as a general rule while the destination is now clear, the path to achieve this is not. Some success has been achieved with inhibitors that have particular conformational biases such as the selectivity of kifunensine for GH47 α-mannosidases. However, the crude attempts to achieve unusual conformations by structural means such as the introduction of bridges across the molecule are typically not tolerated by glycosidase active sites, although the 3,6-anhydrosugars processed by the GH117 α-l-neoagarobiose might constitute a logical target for applying such an approach. More generally, smarter, less intrusive ways are needed to control the conformation of inhibitors through the application of stereoelectronic principles and hybridization. A better understanding of the intrinsic conformational preferences of existing glycosidase inhibitors would greatly assist in directing these efforts.

## Conflicts of interest

None declared.

## References and recommended reading

Papers of particular interest, published within the period of review, have been highlighted as:

• of special interest
•• of outstanding interest
