# Supplementary material for: Dissecting conformational contributions to glycosidase catalysis and inhibition
Source: Curr Opin Struct Biol. 2014 Oct;28:1–13. doi: 10.1016/j.sbi.2014.06.003 (PMC4220041; doi:10.1016/j.sbi.2014.06.003)
Supplement: Supplementary file 1 [file mmc1.docx]

**Table S1.** Proposed conformational itineraries for GH families based on enzyme substrate specificity. Representative data used to assign itineraries is given.

| **Family** | **Mechanism** | **Activity** | **Itinerary** | **Representative data** | |
| --- | --- | --- | --- | --- | --- |
| **GH1** | Retaining | β-glucosidase  myrosinase | ^1^*S*_3_ ↔ ^4^*H*_3_/^4^*E*^‡^ ↔ ^4^*C*_1_ | Michaelis-like complexes | ^1,4^*B*/^1^*S*_3_* (1e56, 1v03, 3gnp, 3f5j, 3f5l, 3f5k, 3aht)  ^4^*C*_1_ (3ai0, 3vil, 2o9r)  ^1^*S*_3_ (3ptq)  DNJ in ^1^*S*_3_ (2j77) |
|  |  |  |  | Covalent intermediate | ^4^*C*_1_ (1oin, 2zox, 3gnr, 3ptm, 2rgm, 3aiw, 3air, 1e70, 1e73, 1e4i) |
|  |  |  |  | Transition state mimics | glucotetrazoles in ^4^*H*_3_ (1v08, 2j7b)  glucohydroxymolactam in *^4^H_3_* (2j78)  glucoimidazole in ^4^*H*_3_ (2j7g)  glucoimidazoles in ^4^*E* (2j7d, 2j7f)  DNJ in ^4^*E* (1oim) |
|  |  |  |  | Products | ^4^*C*_1_ (4hz7, 4hz8, 2o9t, 3vij, 2e9m)  ^4^*C*_1_/^4^*E* (2e9l) |
|  |  |  |  | Computation on conformational itinerary [[1](#_ENREF_1)] | |
|  |  | 6-phospho-β-glucosidase | possibly ^5^*S*_1_ ↔ ??^‡^ ↔ ?? | Michaelis-like complexes | ^5^*S*_1_ (4ipn)  ^4^*C*_1_ (4ipn) |
| **GH2** | Retaining | β-mannosidase | ^1^*S*_5_ ↔ *B*_2,5_^‡^ ↔ ^O^*S*_2_ | Michaelis-like complexes | Michaelis in ^1^*S*_5_ (2wbk) |
|  |  |  |  | Transition state mimics | mannoimidazole in *B*_2,5_/^1^*S*_5_ (2vot, 2vqt, 2vr4)  mannoimidazole in *B*_2,5_ (2vmf)  DMJ in *B*_2,5_ (2vl4) |
|  |  | β-galactosidase | ^1^*S*_3_ (?) ↔ ^4^*H*_3_/^4^*E* ^‡^ ↔ ^4^*C*_1_ | Michaelis-like complexes | ^4^*C*_1_ (1jyv, 1jyv, 1jyx, 1jyy, 1jyz) |
|  |  |  |  | Covalent intermediates | ^4^*C*_1_ (1jz2, 1jz3) |
|  |  |  |  | Transition state mimics | galactotetrazole in ^4^*H*_3_/^4^*E* (1jz6)  galactotetrazole in ^4^*E* (3vd7)  galactonolactone in ^4^*H*_3_ (3vdb, 1jz5) |
|  |  |  |  | Products | ^4^*C*_1_ (1jyn, 1jz7, 1jz8, 3ob8) |
|  |  | β-glucosaminidase |  | Michaelis-like complexes | ^4^*C*_1_ (2vzt, 2vzu, 2vzv) |
|  |  |  |  | Product | ^4^*C*_1_ (2vzs) |
| **GH3** | Retaining | β-*N*-acetylglucosaminidase  β-glucosidase | ^1^*S*_3_ (?) ↔ ^4^*H*_3_/^4^*E*^‡^ ↔ ^4^*C*_1_ | Michaelis-like complexes | ^4^*C*_1_ (4gyj, 4gyk, 4gvi, 4iih, 1iex) |
|  |  |  |  | Covalent intermediate | ^4^*C*_1_ (4gvh, 1iev, 1iew) |
|  |  |  |  | Transition state mimics | PUGNAc in ^4^*H*_3_ (3gs6, 3gsm)  PUGNAc in ^4^*E* (3nvd)  PUGNAc in ^4^*E* (2oxn)  glucoimidazole in ^4^*E* (1lq2) |
|  |  |  |  | Products | ^4^*C*_1_ (4gvf, 2x41, 2x42, 1ieq, 4i3g, 4iig) |
| **GH5** | Retaining | cellobiohydrolase  endoglucanase  β-glucosidase  β-endoglycoceramidase | ^1^*S*_3_ ↔ ^4^*H*_3_^‡^ ↔ ^4^*C*_1_ | Michaelis-like complexes | ^1^*S*_3_/^4^*E* (3qho)  ^1^*S*_3_ (1h2j, 4a3h)  ^1,4^*B*/^1^*S*_3_ (3qhn)  ^4^*C*_1_ (3qhm, 1ece, 2osx) |
|  |  |  |  | Covalent intermediates | ^4^*C*_1_ (1h11, 1h2j, 1qi2, 5a3h, 6a3h, 2osy, 2pb1) |
|  |  |  |  | Transition state mimics | cellobioimidazole in ^4^*H*_3_ (2oyl) |
|  |  |  |  | Products | ^4^*C*_1_ (3a3h, 3amg, 3aof, 3azr, 3azs, 3azt, 3n9k, 3ays) |
|  |  | β-xylanase | unknown | Products | ^4^*C*_1_ (2jeq, 3zmr) |
|  |  | β-mannanase | possibly ^1^*S*_5_ ↔ *B*_2,5_/*E*_5_^‡^ ↔ ^O^*S*_2_ | Transition state mimics | isofagomine lactam in *E*_5_ (1uz4) |
| **GH6** | Inverting | cellobiohydrolase  endoglucanase | ^2^*S*_O_ → ^2,5^*B*^‡^ → ^5^*S*_1_ (?) | Michaelis-like complexes | ^2^*S*_O_ (4avo, 4b4f)  *B*_3,O_/^2^*S*_O_*** (1gz1, 1qk2, 1qjw, 4ax6)  ^4^*C*_1_ (2bof, 2bog, 4ax7, 4au0) |
|  |  |  |  | Transition state mimics | isofagomine in ^2,5^*B*/^2^*S*_O_ (1ocn) |
| **GH7** | Retaining | β-glucosidase  cellobiohydrolase | ^4^*E*/^4^*H*_5_ ↔ ^4^*H*_3_/*E*_3_^‡^ ↔ ^4^*C*_1_ | Michaelis-like complexes | ^4^*E* (4c4c)  ^1,4^*B*/^4^*E* (1ovw)  ^4^*C*_1_ (2rg0, 2rfz, 1q2e, 5cel) |
|  |  |  |  | Transition state mimics | cellobioimidazole in *E*_3_ (1z3w) |
|  |  |  |  | Covalent intermediate | ^4^*C*_1_ (4c4d) |
|  |  |  |  | Products | ^4^*C*_1_ (1ojj, 1ojk) |
| **GH8** | Inverting | endoglucanase  β-glucosidase  cellobiohydrolase | possibly ^2^*S*_O_ → ^2,5^*B*^‡^ → ^5^*S*_1_ | Michaelis-like complex | ^2,5^*B*/^2^*S*_O_* (1kwf) |
|  |  |  |  | Product | ^4^*C*_1_ (3qxq) |
|  |  |  |  | Computation on conformational itinerary [[2](#_ENREF_2)] | |
|  |  | β-xylosidase | possibly ^2^*S*_O_ → ^4^*E*/^4^*H*_3_^‡^ → ^4^*C*_1_ | Computational studies on Michaelis complex[[3](#_ENREF_3)] | |
|  |  |  |  | Michaelis-like complex | ^4^*C*_1_ (2b4f) |
|  |  |  |  | Product | ^4^*C*_1_ (1wu6) |
| **GH9** | Inverting | cellobiohydrolase | possibly ^1^*S*_3_ → ^4^*H*_3_^‡^ → ^4^*C*_1_ | Michaelis-like complex | *^4^H_3_*/^4^*E* (1rq5) |
|  |  |  |  | Products | ^4^*C*_1_ (1k72, 1kfg, 4tf4) |
| **GH10** | Retaining | β-xylanase | ^1^*S*_3_ ↔ ^4^*H*_3_^‡^ ↔ ^4^*C*_1_ | Michaelis-like complexes | ^1^*S*_3_ (2d20, 2d24)  ^4^*C*_1_ (1e5n, 3cui) |
|  |  |  |  | Covalent intermediates | ^4^*C*_1_ (2d22, 1exp) |
|  |  |  |  | Transition state mimics | xylobiose lactam oxime in ^4^*H*_3_/^4^*E* (1fh9)  xylobioseimidazole in ^4^*H*_3_/^4^*E* (1fhd)  xylobio-isofagomine lactam in *E*_5_ (1j01)  xylobio-isofagomine lactam in ^4^*H*_5_ (1od8) |
|  |  |  |  | Product | ^4^*C*_1_ (2d23) |
| **GH11** | Retaining | β-xylanase | possible ^2,5^*B*^‡^ transition state | Michaelis-like complex | ^4^*C*_1_/^O^*E* (4hk8) |
|  |  |  |  | Covalent intermediates | ^2,5^*B* (1bvv, 1qh6, 1h4g)  ^2,5^*B*/^5^*S*_1_ (1c5i) |
|  |  |  |  | Products | ^4^*C*_1_ (1xnk)  ^4^*C*_1_/^O^*E* (4hk9, 4hkw)  ^2,5^*B*/^5^*S*_1_ (1h4h) |
| **GH12** | Retaining | β-glucosidase  cellobiohydrolase | ^1^*S*_3_ ↔ ^4^*H*_3_^‡^ (?)↔ ^4^*C*_1_ | Michaelis-like complexes | ^1,4^*B*/^1^*S*_3_ (1uu6, 1w2u)  ^4^*C*_1_ (1uu5, 3amm) |
|  |  |  |  | Covalent intermediate | ^4^*C*_1_ (2nlr) |
|  |  |  |  | Products | ^4^*C*_1_ (3amn, 3amp, 3amq, 3vl9) |
|  |  | β-xylanase | unknown | Michaelis-like complexes | ^4^*C*_1_ (2bwa, 2bwc) |
|  |  |  |  | Product | ^4^*C*_1_ (2jen) |
| **GH13** | Retaining | α-glucosidase | possibly ^1^*S*_3_ ↔ ^4^*H*_3_^‡^ ↔ ^4^*C*_1_ | Michaelis-like complexes | ^4^*C*_1_ (9cgt, 3k8m, 1cxk) |
|  |  |  |  | Covalent intermediate | ^4^*C*_1_ (1cxl) |
|  |  |  |  | Product | ^4^*C*_1_ (2vr5) |
| **GH14** | Inverting | α-glucosidase | unknown | Michaelis-like complexes | ^4^*C*_1_ (2xgb, 2xgi, 1itc, 1j18) |
| **GH15** | Retaining | α-glucoamylase | unknown | Michaelis-like complex | ^4^*C*_1_ (1gai) |
| **GH16** | Retaining | β-glucanase  β-glucosidase | possibly ^1^*S*_3_ ↔ ^4^*E*/^4^*H*_3_^‡^ ↔ ^4^*C*_1_ | Michaelis-like complex | ^4^*C*_1_ (2vh9) |
|  |  |  |  | Transition state mimic | gluconolactone in ^4^*E* (3azz, 2w39) |
|  |  |  |  | Products | ^4^*C*_1_ (4bpz, 4bow, 1zm1, 1uoa)  ^4^*E* (2w39) |
|  |  |  |  | Computation on conformational itinerary [[4](#_ENREF_4)] | |
|  |  | β-agarase  β-porphyranase | unknown | Michaelis-like complexes | ^4^*C*_1_ (4atf) |
|  |  |  |  | Product | ^4^*C*_1_ (3ilf) |
| **GH17** | Retaining | β-glucanase | unknown | Product | ^4^*C*_1_ (4gzi) |
| **GH18** | Retaining | chitinase | ^1^*S*_5_/^1,4^*B* ↔ ^4^*E*^‡^ (?) ↔ ^4^*C*_1_ | Michaelis-like complexes | ^1^*S*_5_/^1,4^*B* (1e6n)  ^1^*S*_5_ (1nh6, 3a4w, 3a4x, 1eib, 1ehn, 3n18, 3b9a) |
|  |  |  |  | Transition state mimics | chitobionolactone in ^4^*H*_5_ (1ur8) |
|  |  |  |  | Covalent intermediate | oxazoline in ^4^*C*_1_ (1e6z) |
|  |  |  |  | Products | ^1^*S*_5_/^1,4^*B* (3n17)  ^1,4^*B* (4hme, 1ffr, 3b9d)  ^4^*C*_1_ (3n15, 3n13) |
|  |  |  |  | Computation on conformational itinerary [[5](#_ENREF_5)] | |
|  |  |  |  | Computation on conformational itinerary [[6](#_ENREF_6)] | |
| **GH19** | Inverting | chitinase | unknown | Product | ^4^*C*_1_ (4jol) |
| **GH20** | Retaining | *β-*hexosaminidase | ^1,4^*B*/^1^*S*_3_ (?) ↔ ^4^*E*/^4^*H*_3_^‡^ ↔ ^4^*C*_1_ | Michaelis-like complexes | ^1,4^*B* (1c7s, 2yl9)  ^4^*E*/^1,4^*B* (1c7t, 1qbb, 2yl8, 2yla) |
|  |  |  |  | Covalent intermediates | oxazoline in ^4^*C*_1_ (4c7g)  thiazoline in ^4^*C*_1_ (4jaw, 3sus) |
|  |  |  |  | Transition state mimics | LOGNAc in ^4^*E* (4az7, 4azh)  PUGNAc in ^4^*E* (4azb, 4az6, 4azg, 4azi)  GlcNAc lactone in ^4^*E* (1o7a) |
|  |  |  |  | Products | ^4^*C*_1_ (1m01, 1m03, 1m04)  ^1,4^*B* (1m03)  ^4^*E*/^4^*H*_5_ (4h04) |
|  |  |  |  | Extensive analysis of structures of GH20 at <http://www.jbc.org/content/288/17/11795/suppl/DC1> | |
|  |  |  |  | Computation on conformational itinerary [[7](#_ENREF_7)] | |
| **GH22** | Retaining | lysozyme | ^1^*S*_3_ (?) ↔ ^4^*H*_3_^‡^ ↔ ^4^*C*_1_ | Covalent intermediate | ^4^*C*_1_ (1h6m) |
|  |  |  |  | Transition state mimic | tetra-*N*-acetyl chitotetraose lactone in *E*_3_/^4^*H*_3_[[8](#_ENREF_8)] |
|  |  |  |  | Products | *E*_3_/^4^*H*_3_[[9](#_ENREF_9)]  ^4^*C*_1_ (1lmq, 1ljn, 1lzr, 1lzs, 1lzb, 1lzc, 1lze) |
| **GH23** | Inverting | chitinase  lysozyme | unknown | Michaelis-like complexes | ^4^*C*_1_ (3w6d) |
|  |  |  |  | Products | ^1,4^*B* (4kpm)  ^4^*C*_1_ (154l, 3w6c, 3w6f, 3gxr) |
|  | Retaining | lytic transglycosidase | unknown | Michaelis-like complexes | ^4^*C*_1_ (4hjy, 4hjz) |
|  |  |  |  | Product | ^4^*C*_1_ (3bkv) |
| **GH24** | Inverting | lysozyme | unknown |  |  |
| **GH26** | Retaining | β-mannanase | ^1^*S*_5_ ↔ *B*_2,5_^‡^ ↔ ^O^*S*_2_ | Michaelis-like complex | ^1^*S*_5_ (2vx6, 1gwy) |
|  |  |  |  | Covalent intermediate | ^O^*S*_2_ (1gw1) |
|  |  |  |  | Transition state mimic | mannobioimidazole in *B*_2,5_ (4cd5) |
|  |  |  |  | Products | ^4^*C*_1_ (2vx7, 2whm, 1odz) |
|  |  | lichenases  β-1,3-xylanase | ^1^*S*_3_ ↔ ^4^*H*_3_^‡^ (?) ↔ ^4^*C*_1_ | Michaelis-like complex | ^1,4^*B*/^1^*S*_3_ (2cip) |
|  |  |  |  | Covalent intermediates | ^4^*C*_1_ (2cit, 3vpl) |
|  |  |  |  | Product | ^4^*C*_1_ (2vi0) |
| **GH27** | Retaining | α-galactosidase  β-L-arabinopyranosidase  α-*N*-acetylgalactosaminidase | ^4^*C*_1_ ↔ *E*_3/_^4^*H*_3_^‡^ (?) ↔ ^1^*S*_3_ | Michaelis-like complex | ^4^*C*_1_ (3hg3) |
|  |  |  |  | Covalent intermediates | ^1^*S*_3_ (3hg4, 3igu) |
|  |  |  |  | Products | ^4^*C*_1_ (3a22, 3hg5, 3s5z, 3h54, 3h55, 1r47, 3gxt) |
|  |  |  |  | Computation on conformational itinerary[[10](#_ENREF_10)] | |
| **GH28** | Inverting | polygalacturonases | unknown | Products | ^4^*C*_1_ (1kcc, 2uvf) |
| **GH29** | Retaining | α-fucosidase | ^1^*C*_4_ ↔ ^3^*H*_4_^‡^ (?) ↔ ^3^*S*_1_ | Michaelis-like complexes | ^1^*C*_4_ (3uet, 2wvu) |
|  |  |  |  | Covalent intermediates | ^3^*S*_1_ (2wvs)  ^1^*C*_4_/^3^*S*_1_ (1hl9) |
|  |  |  |  | Transition state mimic | pyrrolidine in ^3^*E* (4j28) |
|  |  |  |  | Product | ^1^*C*_4_ (1odu) |
| **GH30** | Retaining | β-glucosidase  glucuronoxylan xylanohydrolases | possibly ^1^*S*_3_/^1^*S*_5_ ↔ ^4^*E*/^4^*H*_3_^‡^ ↔ ^4^*C*_1_ | Michaelis-like complex | isofagomine in ^1^*S*_5_ (3gxf) |
|  |  |  |  | Transition state mimic | glucoamidine in ^4^*E* (2xwd, 2xwe) |
|  |  |  |  | Products | ^4^*C*_1_ (3kl5, 2y24) |
| **GH31** | Retaining | α-glucosidase  α-xylosidase | possibly ^4^*C*_1_↔ ^4^*H*_3_^‡^ (?) ↔^1^*S*_3_ | Michaelis-like complex | ^4^*C*_1_ (2f2h) |
|  |  |  |  | Covalent intermediates | ^1^*S*_3_ (4ba0, 1xsk, 2xvk) |
| **GH32** | Retaining | β-fructofuranosidase | probable ^3^*E*^‡^ | Michaelis-like complexes | ^3^*E* (4ffh, 4ffi, 4ffg)  ^3^*T*_2_ (1w2t, 2aez, 3ui4, 3u75)  *E*_2_ (3lih, 2qqu, 2qqv, 2qqw) |
|  |  |  |  | Transition state mimic | pyrrolidine in ^3^*E* (2aey) |
|  |  |  |  | Products | *E*_2_ (3pij, 3kf3)  ^3^*T*_2_ (2xqr) |
| **GH33** | Retaining | α-sialidase | ^6^*S*_2_ ↔ ^4^*H*_5_^‡^ ↔ ^2^*C*_5_ | Michaelis-like complexes | *B*_2,5_ (1s0i)  ^6^*S*_2_ (1s0j) |
|  |  |  |  | Covalent intermediates | ^2^*C*_5_ (2vk7, 2xzk, 4nc5, 4ncs, 2a75, 2ags, 2fhr, 2ah2) |
|  |  |  |  | Transition state mimic | oseltamivir in ^4^*H*_5_ (2ya8) |
|  |  |  |  | Product-like complexes | ^2^*C*_5_ (2ber, 1w0p)  ^4^*S*_2_ (2ya5, 3h72)  *B*_2,5_ (2xzi)  *E*_5_ (2bf6, 1n1y) |
| **GH34** | Retaining | neuraminidase | ^4^*S*_2_*/*^6^*S*_2_↔ ^4^*H*_5_^‡^ ↔ ^2^*C*_5_ | Michaelis-like complexes | ^6^*S*_2_/^6^*H*_5_ (4gzx) |
|  |  |  |  | Covalent intermediates | ^2^*C*_5_ (4h52, 3w09) |
|  |  |  |  | Transition state mimics | oseltamivir in ^4^*H*_5_ (2ht7, 2ht8, 2hu4, 4k1i, 2qwh, 3cl2) |
|  |  |  |  | Products | ^4^*S*_2_ (4gzq, 2qwb)  *E*_5_/*B*_2,5_ (4gzw)  *E*_5_ (1nsc)  ^2^*C*_5_ (4h53, 1mwe) |
| **GH35** | Retaining | β-galactosidase | unknown | Products | ^4^*C*_1_ (3ogr, 1xc6, 4e8c, 4iug, 3thc) |
| **GH36** | Retaining | α-galactosidase | unknown | Michaelis-like complexes | ^4^*C*_1_ (4fnt, 4fnu) |
|  |  |  |  | Product | ^4^*C*_1_ (2xn2, 2yfo) |
| **GH37** | Inverting | trehalase | unknown |  |  |
| **GH38** | Retaining | α-mannosidase | ^O^*S*_2_↔ *B*_2,5_^‡^ ↔ ^1^*S*_5_ | Michaelis-like complexes | ^4^*C*_1_ (3bvt, 3bvu, 3bvv, 3bvw, 3bvx, 3cv5, 3czn) |
|  |  |  |  | Covalent intermediates | ^1^*S*_5_ (1qwn, 1qwu, 1qx1) |
|  |  |  |  | Transition state mimic | mannoimidazole in *^4^H_3_* (3d4y) – later ^4^*H*_3_ and *B*_2,5_ [[11](#_ENREF_11)] |
|  |  |  |  | Product-like complexes | ^4^*C*_1_ (3czs, 3buq)  ^O^*H*_5_ (3bup)  noeuromycin in ^1^*S*_5_ (2alw) |
|  |  |  |  | Computation on conformational itinerary[[12](#_ENREF_12)] | |
| **GH39** | Retaining | β-xylosidase | ^1^*S*_3_ ↔ *^4^H_3_*^‡^ (?) ↔^4^*C*_1_ | Michaelis-like complexes | ^1^*S*_3_ (2bfg) |
|  |  |  |  | Covalent intermediate | ^4^*C*_1_ (2bfg, 1uhv) |
|  |  |  |  | Product | ^4^*C*_1_ (1px8) |
|  |  | α-idurodinase | ^2^*S*_O_ ↔ ^2,5^*B* ^‡^ (?) ↔^5^*S*_1_ | Michaelis-like complexes | ^2^*S*_O_ (4kgj)  ido-DNJ-type in ^2^*S*_O_ (4kgl) |
|  |  |  |  | Covalent intermediate | ^5^*S*_1_ (4kh2) |
| **GH42** | Retaining | β-galactosidase | unknown | Product | ^4^*C*_1_ (3tty, 1kwk) |
| **GH43** | Inverting | *β*-galactanase | ^1^*S*_3_ ↔ ^4^*H*_3_^‡^ (?) ↔ ^4^*C*_1_ | Michaelis-like complexes | ^4^*C*_1_ (3vsz, 3vt0)  ^1^*S*_3_ (3vt2) |
|  |  |  |  | Product | ^4^*C*_1_ (3vt1) |
|  |  | *β*-xylosidase | unknown | Michaelis-like complexes | ^2,5^*B*/^2^*S*_O_ (2exj, 2exk)  ^2^*S*_O_ (2exj)  ^2,5^*B* (2exk)  ^4^*C*_1_ (2exj, 2exk) |
|  |  | α-arabinofuranosidase | unknown | Michaelis-like complexes | ^2^*T*_1_ (3d5z)  *E*_2_ (1gye)  ^4^*T*_O_ (3qef) |
|  |  |  |  | Products | *E*_2_ (3akg)  ^2^*E* (3akh)  ^1^*T*_O_ (3aki) |
| **GH44** | Retaining | endoglucanase  xyloglucanase | possibly ^1^*S*_3_ ↔ ^4^*H*_3_^‡^ (?) ↔ ^4^*C*_1_ | Michaelis-like complexes | ^1^*S*_3_ (2eqd) |
|  |  |  |  | Products | ^4^*C*_1_ (2eex, 2ej1)  ^1^*S*_5_ (2e0p, 2eo7)  ^1^*S*_3_ (3ii1) |
| **GH45** | Inverting | endoglucanase | unknown | Product | ^4^*C*_1_ (4eng) |
| **GH46** | Inverting | endoglucanase | unknown | Product | ^4^*C*_1_ (1qgi) |
| **GH47** | Inverting | α-mannosidase | ^3,O^*B/*^3^*S*_1_→ ^3^*H*_4_^‡^ → ^1^*C*_4_ | Michaelis-like complexes | ^3,O^*B/*^3^*S*_1_ (4ayp, 1x9d)  ^1^*C*_4_ (2ri9) |
|  |  |  |  | Transition state mimics | mannoimidazole in ^3^*E*/^3^*H*_4_ (4ayq) |
|  |  |  |  | Product-like complex | noeuromycin in ^1^*C*_4_ (4ayr) |
| **GH48** | Inverting | cellobiohydrolase | unknown | Michaelis-like complexes | ^4^*C*_1_ (1g9j, 2qno) |
|  |  |  |  | Products | ^4^*C*_1_ (1f9d, 1fbw) |
| **GH50** | Retaining | β-agarase | unknown | Michaelis-like complexes | ^1^*S*_3_ (4bq4, 4bq5) |
| **GH51** | Retaining | α-arabinofuranosidase | ^4^*E /*^4^*T*_O_ ↔ *E*_3_(?) ^‡^ ↔ ^2^*T*_1_/^2^*E* | Michaelis-like complexes | ^2^*E* (2c7f)  ^4^*E* (1qw9, 2vrq)  ^4^*T*_O_ (2c8n, 1qw8, 1qw9) |
|  |  |  |  | Covalent intermediates | ^2^*T*_1_ (1pz2)  ^2^*E* (1pz2) |
|  |  |  |  | Product | ^4^*T*_3_ (3ug4) |
| **GH54** | Retaining | α-arabinofuranosidase | unknown | Product | *E*_3_ (1wd4) |
| **GH55** | Inverting | β-glucanase | possibly ^1^*S*_3_ → *E*_3_/^4^*H*_3_^‡^ → ^4^*C*_1_ | Transition state mimics | gluconolactone in ^4^*H*_3_ (3eqo)  gluconolactone in ^1^*S*_3_*/E*_3_ (3eqo) |
| **GH56** | Retaining | β-hyaluronidase | unknown | Product | ^1,4^*B*/^1^*S*_5_*(1fcv) |
| **GH57** | Retaining | α-glucanotransferase | unknown |  |  |
| **GH58** | Inverting | endosialidase | unknown | Products | ^2^*C*_5_ (1v0f)  ^4^*H*_5_/*E*_5_ (3gvk)  ^2^*C*_5_/*E*_5_ (3gvk) |
| **GH59** | Retaining | β-galactocerebrosidase | unknown | Michaelis-like complex | ^4^*C*_1_ (4ccc) |
|  |  |  |  | Covalent intermediate | ^4^*C*_1_ (4ccd) |
|  |  |  |  | Product | ^4^*C*_1_ (4cce, 3zr6) |
| **GH62** | Inverting (?) | α-arabinofuranosidase | unknown | Product | ^2^*E* (4n2r) |
| **GH63** | Inverting | α-glucosidase | unknown | Michaelis-like complexes | ^4^*C*_1_ (3w7w, 3w7x) |
| **GH65** | Inverting | kojibiose phosphorylase | unknown | Michaelis-like complex | ^4^*C*_1_ (3wiq) |
|  |  |  |  | Product | ^4^*C*_1_ (3wir) |
| **GH66** | Retaining | dextranase | unknown | Product | ^4^*C*_1_ (3vmo) |
| **GH67** | Inverting | α-glucuronidase | unknown | Michaelis-like complexes | *^2^E* (1k9f) |
|  |  |  |  | Products | *^2^E* (1k9e)  ^4^*C*_1_ (1l8n, 1mqq, 1h41,1gqk, 1gql) |
| **GH68** | Retaining | β-fructofuranosidase  levansucrase | unknown | Michaelis-like complexes | *E*_2_ (1pt2, 2yfs, 2yft) |
|  |  |  |  | Product | *E*_2_ (3vss) |
| **GH70** | Retaining | glucansucrase | unknown |  |  |
| **GH72** | Retaining | β-glucanase | unknown | Product | ^4^*C*_1_ (2w62) |
| **GH74** | Inverting | β-xyloglucanase | ^1^*S*_3_→ ^4^*H*_3_^‡^ (?) →*^4^C_1_* | Michaelis-like complex | ^1^*S*_3_ (2ebs) |
|  |  |  |  | Product | ^4^*C*_1_ (2cn3) |
| **GH77** | Retaining | α-amylomaltase | unknown | Michaelis-like complex | ^4^*C*_1_ (1esw) |
|  |  |  |  | Covalent intermediates | ^4^*C*_1_ (2owc, 2oww) |
| **GH78** | Retaining | α-rhamnosidase | possibly ^2^*S*_O_ ↔ ^2,5^*B* ^‡^ ↔^5^*S*_1_ | Product | ^5^*S*_1_ (3w5n) |
| **GH79** | Retaining | β-glucuronidase | unknown | Covalent intermediate | ^4^*C*_1_ (3vo0) |
|  |  |  |  | Product | ^4^*C*_1_ (3vnz) |
| **GH83** | Retaining(?) | neuraminidase | unknown | Michaelis-like complex | *B*_2,5_/*E*_5_ (1z4x) |
|  |  |  |  | Products | ^2^*C*_5_ (1v3c, 1e8u) |
| **GH84** | Retaining | β-glucosaminidase  hyaluronidase | ^1^*S*_3_ ↔ ^4^*H*_3_/^4^*E*^‡^ ↔ ^4^*C*_1_ | Michaelis-like complex | ^4^*E*/^1^*S*_3_ (2x0h) |
|  |  |  |  | Transition state mimics | PUGNAc-imidazole in ^4^*E* (2j47, 2j62, 2wb5)  PUGNAc in ^4^*E* (2cbj)  GlcNAc lactam in ^4^*E* (2xm1)  GlcNAc lactone in ^4^*E* (2xsb)  LOGNAc in ^4^*E* (2xm2) |
|  |  |  |  | Covalent intermediates | oxazoline in ^4^*C*_1_ (2wzh)  5F-oxazoline in ^4^*C*_1_ (2wzi) |
| **GH85** | Retaining | β-glucosaminidase | unknown | Covalent intermediate | NAGthiazoline in ^4^*C*_1_ (2w92) |
| **GH86** | Retaining(?) | β-porphyrinase | unknown | Product | ^4^*C*_1_ (4aw7) |
| **GH88** | Not Applicable | unsaturated glucuronyl hydrolase | unknown | Michaelis-like complexes | ^2^*H*_1_ (2ahg)  ^2^*H*_1_ (2fv0, 2fv1)  ^2^*E* (3ank) |
| **GH 89** | Retaining | α-*N*-acetyl-glucosaminidase | unknown | Michaelis-like complex | ^4^*C*_1_ (4a4a) |
|  |  |  |  | Product | ^4^*C*_1_ (2vca) |
| **GH 90** | Inverting (?) | α-rhamnosidase | possibly ^5^*S*_1_→ ^2,5^*B* ^‡^ →^2^*S*_O_ | Michaelis-like complexes | ^2,5^*B*/^5^*S*_1_ (1tyu, 1tyw, 1tyx)  ^5^*E* (3tho) |
| **GH 91** | Inverting (?) | β-fructotransferase | unknown | Michaelis-like complex | ^1^*T*_2_ (2inv) |
| **GH 92** | Inverting | α-mannosidase | ^O^*S*_2_ (?)→ *B*_2,5_^‡^ → ^1^*S*_5_ | Michaelis-like complexes | ^4^*C*_1_ (2ww1, 2ww3) |
|  |  |  |  | Transition state mimics | mannoimidazole in ^1^*S*_5_/*B*_2,5_ (2wzs) |
|  |  |  |  | Product-like complex | kifunensine in ^1,4^*B*/^1^*S*_5_ (2wvz) |
| **GH 93** | Inverting | α-arabinofuranosidase | unknown | Michaelis-like complex | pyrrolidine in ^4^*T*_O_ (2ydp, 2ydt) |
|  |  |  |  | Products | *E*_3_ (2w5o)  ^4^*T*_3_ (3a72) |
| **GH 94** | Inverting | cellobiose phosphorylase | possibly ^1^*S*_3_→ ^4^*H*_3_^‡^ → ^4^*C*_1_ | Michaelis-like complexes | DNJ in ^1^*S*_3_*/ B*_3,O_ (3qg0)  DNJ in ^4^*C*_1_ (3qfy, 3qfz, 3qg0) |
|  |  |  |  | Product | ^4^*C*_1_ (2cqs, 2cqt) |
| **GH 95** | Inverting | α-fucosidase | unknown | Michaelis-like complex | ^1^*C*_4_ (2ead) |
|  |  |  |  | Product | ^1^*C*_4_ (2eae) |
| **GH 97** | Inverting and Retaining | α-glucosidase  (Inverting) | unknown |  |  |
| **GH 98** | Inverting | α-galactosidase | unknown | Michaelis-like complex | ^4^*C*_1_*/*^4^*E* (2wmk, 2wmg) |
|  |  |  |  | Products | ^4^*C*_1_ (2wmj, 2wmh, 2wmi) |
| **GH 99** | Retaining | endo-α-mannosidase | possibly ^4^*C*_1_↔ ^4^*E*^‡^ ↔ ^4^*H*_5_ | Itinerary a consequence of the proposed mechanism proceeding through a 1,2-anhydro sugar in a ^4^*H*_5_ conformation[[13](#_ENREF_13)] | |
| **GH102** | Retaining | lytic transglycosylase | unknown | Product | ^4^*C*_1_ (2pi8) |
| **GH103** | Retaining | lytic transglycosylase | unknown | Product | ^4^*C*_1_ (1d0k) |
| **GH104** | Retaining | lytic transglycosylase | unknown | Product | ^4^*C*_1_ (1d9u, 3d3d) |
| **GH105** | Retaining | unsaturated rhamnogalacturonyl hydrolase | unknown | Michaelis-like complexes | *E*_2_/*B*_2,5_ (2d8l)  ^2^*H*_1_/^2^*E* (2gh4) |
| **GH 112** | Inverting | galacto-*N*-biose phosphorylase | unknown | Products | ^4^*C*_1_ (2zut, 2zuu, 2zuv, 2zuw) |
| **GH 113** | Retaining | β-mannosidase | ^1^*S*_5_ ↔ *B*_2,5_^‡^ ↔ ^O^*S*_2_ | Transition state mimic | mannobioimidazole in *B*_2,5_ (4cd8) |
| **GH 117** | Inverting | α-agarase | possibly ^1,4^*B* → ^4^*E* → ^4^*C*_1_ | Michaelis-like complex | ^1,4^*B* (4ak7) |
| **GH120** | Retaining | β-xylosidase | possibly ^2^*S*_O_ ↔ ^2,5^*B* ^‡^ ↔ ^5^*S*_1_ | Michaelis-like complex | ^2,5^*B*/^2^*S*_O_* (3vsu) |
|  |  |  |  | Product | ^2^*S*_O_ (3vsv) |
| **GH 125** | Inverting | α-mannosidase | unknown | Michaelis-like complexes | ^4^*C*_1_ (3qt9, 3qsp) |
| **GH 130** | Inverting | β-1,4-mannosylglucose phosphorylase | possibly ^1^*S*_5_ → *B*_2,5_^‡^ → ^O^*S*_2_ | Michaelis-like complex | ^1^*S*_5_ (3was) |
|  |  |  |  | Products | mannose in ^O^*S*_2_ (3wat)  ^4^*C*_1_ (3wau) |

*=structures revised/commented upon in ref [[14](#_ENREF_14)].

**References**

1. Badieyan S, Bevan DR, Zhang C: **Probing the active site chemistry of beta-glucosidases along the hydrolysis reaction pathway**. *Biochemistry* 2012, **51**:8907-8918.

2. Petersen L, Ardevol A, Rovira C, Reilly PJ: **Mechanism of cellulose hydrolysis by inverting GH8 endoglucanases: a QM/MM metadynamics study**. *J Phys Chem B* 2009, **113**:7331-7339.

3. De Vos D, Collins T, Nerinckx W, Savvides SN, Claeyssens M, Gerday C, Feller G, Van Beeumen J: **Oligosaccharide binding in family 8 glycosidases: crystal structures of active-site mutants of the beta-1,4-xylanase pXyl from Pseudoaltermonas haloplanktis TAH3a in complex with substrate and product**. *Biochemistry* 2006, **45**:4797-4807.

4. Biarnes X, Ardevol A, Iglesias-Fernandez J, Planas A, Rovira C: **Catalytic itinerary in 1,3-1,4-beta-glucanase unraveled by QM/MM metadynamics. Charge is not yet fully developed at the oxocarbenium ion-like transition state**. *J Am Chem Soc* 2011, **133**:20301-20309.

5. Jitonnom J, Lee VS, Nimmanpipug P, Rowlands HA, Mulholland AJ: **Quantum mechanics/molecular mechanics modeling of substrate-assisted catalysis in family 18 chitinases: conformational changes and the role of Asp142 in catalysis in ChiB**. *Biochemistry* 2011, **50**:4697-4711.

6. Brameld KA, Goddard WA: **Substrate Distortion to a Boat Conformation at Subsite −1 Is Critical in the Mechanism of Family 18 Chitinases**. *Journal of the American Chemical Society* 1998, **120**:3571-3580.

7. Passos O, Fernandes PA, Ramos MJ: **QM/MM study of the catalytic mechanism of GalNAc removal from GM2 ganglioside catalyzed by human beta-hexosaminidaseA**. *J Phys Chem B* 2011, **115**:14751-14759.

8. Ford LO, Johnson LN, Machin PA, Phillips DC, Tjian R: **Crystal structure of a lysozyme-tetrasaccharide lactone complex**. *J Mol Biol* 1974, **88**:349-371.

9. Strynadka NC, James MN: **Lysozyme revisited: crystallographic evidence for distortion of an N-acetylmuramic acid residue bound in site D**. *J Mol Biol* 1991, **220**:401-424.

10. Pan XL, Liu W, Liu JY: **Mechanism of the glycosylation step catalyzed by human alpha-galactosidase: a QM/MM metadynamics study**. *J Phys Chem B* 2013, **117**:484-489.

11. Williams RJ, Iglesias-Fernández J, Stepper J, Jackson A, Thompson AJ, Lowe EC, White JM, Gilbert HJ, Rovira C, Davies GJ, et al.: **Combined Inhibitor Free-Energy Landscape and Structural Analysis Reports on the Mannosidase Conformational Coordinate**. *Angewandte Chemie International Edition* 2014, **53**:1087-1091.

12. Petersen L, Ardèvol A, Rovira C, Reilly PJ: **Molecular Mechanism of the Glycosylation Step Catalyzed by Golgi α-Mannosidase II: A QM/MM Metadynamics Investigation**. *Journal of the American Chemical Society* 2010, **132**:8291-8300.

13. Thompson AJ, Williams RJ, Hakki Z, Alonzi DS, Wennekes T, Gloster TM, Songsrirote K, Thomas-Oates JE, Wrodnigg TM, Spreitz J, et al.: **Structural and mechanistic insight into N-glycan processing by endo-α-mannosidase**. *Proceedings of the National Academy of Sciences* 2012, **109**:781-786.

14. Biarnes X, Ardevol A, Planas A, Rovira C, Laio A, Parrinello M: **The conformational free energy landscape of beta-D-glucopyranose. Implications for substrate preactivation in beta-glucoside hydrolases**. *J Am Chem Soc* 2007, **129**:10686-10693.
